# A Metasynthesis and Meta-analysis of the Impact and Diagnostic Safety of COVID-19 Symptom Agnostic Rapid Testing in Low- and Middle-Income Countries: Protocol for a Systematic Review

**DOI:** 10.2196/41132

**Published:** 2023-01-05

**Authors:** Mathew Mbwogge, Pratyush Kumar, Kumar Abhishek

**Affiliations:** 1 See Acknowledgments; 2 Dr Baba Saheb Ambedkar Medical College and Hospital New Delhi India

**Keywords:** SARS-CoV-2, 2019-nCoV, rapid test, diagnostic accuracy, meta-analysis, metasynthesis, COVID-19, low- and middle-income countries

## Abstract

**Background:**

Amid all public health measures to contain COVID-19, the most challenging has been how to break the transmission chain. This has been even more challenging in low- and middle-income countries (LMICs). A public health emergency warrants a public health perspective, which comes down to prevention. Rapid mass testing has been advocated throughout the pandemic as a way to promptly deal with asymptomatic infections, but its usefulness in LMICs is yet to be fully understood.

**Objective:**

The study objectives of this paper are to (1) investigate the impact of the different rapid mass testing options for SARS-CoV-2 that have been delivered at point of care in LMICs and (2) evaluate the diagnostic safety (accuracy) of rapid mass testing for SARS-CoV-2 in LMICs.

**Methods:**

This review will systematically search records in PubMed, EBSCOhost, Cochrane library, Global Index Medicus COVID-19 Register, and Scopus. Records will be managed using Mendeley reference manager and SWIFT-Review. Risk of bias for randomized controlled trials will be assessed using the RoB 2 assessment tool, while nonrandomized interventions will be assessed using the tool developed by the Evidence Project. A narrative approach will be used to synthesize data under the first objective, and either a meta-analysis or synthesis without meta-analysis for the second objective. Tables, figures, and textual descriptions will be used to present findings. The overall body of evidence for the first objective will be assessed using the Grading of Recommendations Assessment, Development, and Evaluation–Confidence in the Evidence from Reviews of Qualitative research (GRADE-CERQual) approach, and for the second objective using GRADE.

**Results:**

The screening of records has been finalized. We hope to finalize the synthesis by the end of February 2023 and to prepare the manuscript for publication by April 2023. The study will be reported in accordance with standard guidelines for the reporting of systematic reviews. Review results will be disseminated through conferences and their peer-reviewed publication in a relevant journal.

**Conclusions:**

This review highlights the role of a preventive approach in infection control using rapid mass testing. It also flags the overriding need to involve users and providers in the evaluation of such tests in the settings for which they are intended. This will be the first review to the best of our knowledge to generate both qualitative and quantitative evidence regarding rapid mass testing specific to LMICs.

**Trial Registration:**

PROSPERO CRD42022283776; https://www.crd.york.ac.uk/prospero/display_record.php?RecordID=283776

**International Registered Report Identifier (IRRID):**

PRR1-10.2196/41132

## Introduction

### Background

The real challenge of COVID-19 containment is to break the transmission chain by preventing individuals from getting infected and preventing those infected from transmitting to others [1]. The gold standard of COVID-19 containment has shifted to mass vaccination, with the belief that vaccines will break the chain of transmission. It is reported that current vaccines are close to 100% effective in reducing hospitalizations and deaths [2]. These significant efficacy results from conducted studies may however be limited given that these are just 2 of the endpoints of vaccine effectiveness. In reality, the effectiveness of available vaccines in preventing *infections* is yet to be fully understood in many countries. The duration of immunity of current vaccines is also a critical yet unanswered question [3]. It becomes even more challenging to measure the above endpoints amid emerging new variants [4-8] since this requires constant knowledge renewal on viral genomic epidemiology [9]. New variants of SARS-CoV-2, including B.1.1.7, B.1.351, and P.1, have been reported in the United Kingdom, South Africa, and Brazil, respectively. The emergence of these new variants—when the global economy is yet to recover from the devastating effects of the previous variants still in circulation—is particularly challenging. More so, the fact that these new variants of concern [10,11] are not only more transmissible but appear to be more asymptomatic and resistant to polymerase chain reaction detection, flags the overriding importance of waiting time in the detection of those infected. Transmissibility of the virus appears to vary by context as factors reported to determine transmission include the capacity for the virus to replicate, the exhibition of symptoms (cough), and the individual associated environmental factors (including behavior) [12].

It is reported that a major factor that can contribute to up to 50% of infected persons being overlooked during symptom screening is the inability to detect the virus during the asymptomatic stage by programs designed to diagnose cases when the disease is quite advanced [13]. Apart from a group of people who deliberately might not declare they have symptoms, the first 2 days of the 12-day infectious period of the virus, during which infected persons do not show any symptoms, remain an important proportion in the transmission mechanism of a deadly disease. The inability to effectively manage the transmission mechanism in low- and middle-income countries (LMICs) due to limited resources prompts the need for low-cost rapid tests in these countries. While some remain skeptical about the diagnostic safety and effectiveness of rapid tests [14,15], others believe that the currently available rapid tests are equally accurate enough in detecting some of the new variants [16-18]. The current evidence of what these different tests are and their diagnostic safety [19] within the context of LMIC is limited. In addition, standard guidelines for their use [20,21] seldom exist in these settings, making their effective deployment more challenging.

Key questions to ask when planning to identify infections promptly include, among others, the following: (1) When to test? (2) Where to test? (3) When to get the results? (4) How safe? and (5) How regular? As the global economy learns to cope with the more silent new variant infections, it is apparent that low-cost regular rapid mass testing might assume an important role [22,23], but their diagnostic safety [24] may be an issue particularly in LMICs. According to a 2020 report by the World Bank, low-income countries are those with a gross national income per capita of ≤US $1045, and middle-income countries are those with a gross national income per capita of US $1046 to US $12,695 [25]. About 500 million tests have been estimated to be needed in LMICs in 2021 [26], and the adoption of low-cost rapid tests might lead to up to 50% cost savings [27,28]; however, their fitness for purpose in these countries is yet to be fully understood. This study seeks to systematically review the evidence-based rapid mass testing options for SARS-CoV-2 that can be delivered at point of care in LMICs.

### Specific Objectives

The specific objectives of this study are as follows: (1) to investigate the impact of the different rapid mass testing options for SARS-CoV-2 that have been delivered at point of care in LMICs and (2) to evaluate the diagnostic accuracy of rapid mass testing for SARS-CoV-2 in LMICs.

### Research Questions

Given that our review was geared toward going beyond a quantitative evidence synthesis, the research questions were framed following the Perspective, Setting, Phenomenon, Environment, Comparison, Timing, and Findings (PerSPEcTiF) framework to incorporate the (1) setting, (2) time, and (3) place [29] for objective 1, and using the population, intervention, comparison, outcome (PICO) approach for objective 2 [30].

#### Objective 1

From a preventive health perspective in a LMIC, what do rapid tests for SARS-CoV-2—in an environment of limited resources with silent transmissions from infection up until diagnosis—mean to patients, health personnel, and the general public?

#### Objective 2

What is the diagnostic accuracy of near-the-patient rapid mass testing options for SARS-CoV-2 implemented, irrespective of symptoms in LMICs?

## Methods

### Study Design

This is a systematic review of both qualitative and quantitative evidence synthesis. A qualitative approach will take the form of a metasynthesis while the quantitative approach will take the form of a meta-analysis.

### Eligibility Criteria

Searched records will be screened using the inclusion and exclusion criteria. Given the important role played by both contextual variables and time in the detection of SARS-CoV-2 to break the transmission chain, we believed that rapid mass testing interventions were complex ones [31], not only following a systems approach but underpinned by both contextual variability and time scale [29]. The eligibility criteria for the first objective follows the PerSPEcTiF framework, while that for the second objective follows the PICO framework, as seen in Table 1.

**Table 1:** Inclusion and exclusion criteria.

Objective		Inclusion	Exclusion
**Objective 1 (PerSPEcTiF^a^)**			
	Perspective	Preventive care [32,33] and public health [21,34] perspective	Diseased population
	Setting	Point of care or near the patient [35,36], including GP^b^ surgeries; physician offices; community (population); walk-in or drive-through testing sites; primary care centers; nursing; and long-term care facilities, hospital wards, clinics, prisons, pharmacies, schools, workplaces, sports centers, and border testing sites	Lab-based testing
	Phenomenon	Non–lab-based rapid and point of care test [37]; they are highly accessible, applied irrespective of symptoms, easily transported, and inexpensive, and should provide results in 1 visit [38].	Serology tests, lab-based tests, and non–point of care tests
	Environment	In low- and middle-income countries [25]	High-income countries
	Comparison	N/A^c^	N/A
	Timing	Time during which infection remains asymptomatic; results delivered in 1 visit	Time of diagnosis includes symptoms; if time to results is more than 24 hours
	Findings	Impact of rapid tests including the knowledge, attitude, and perception of patients, health care providers, and the public	Studies not evaluating the impact of rapid tests on the public
**Objective 2 (PICO^d^)**			
	Participants	Asymptomatic patients with COVID-19 and close contacts of index cases	COVID-19 or symptomatic patients
	Intervention	Rapid antigen and point-of-care tests	Lab-based tests
	Comparison	Lab-based reverse transcription–polymerase chain reaction [39-41]; the test could miss up to 30%-40% of infections [42,43]	Tests other than reverse transcription polymerase chain reactions
	Outcome	Diagnostic test accuracy	Nondiagnostic test accuracy studies
**Other**			
	Studies	Qualitative and mixed methods studies for objective 1; RCTs^e^, non-RCTs, and cross-sectional cohort studies for objective 2	Longitudinal cohort, case-control, case-control cross-sectional, and all prognostic and predictive DTA^f^ studies

^a^PerSPEcTiF: Perspective, Setting, Phenomenon, Environment, Comparison, Timing, and Findings.

^b^GP: general practitioner.

^c^N/A: not applicable.

^d^PICO: population, intervention, comparison, outcome.

^e^RCT: randomized controlled trials.

^f^DTA: diagnostic test accuracy.

### Information Sourcing

#### Electronic Databases

The literature search will be performed in PubMed, EBSCOhost (CINAHL Plus), Global Index Medicus COVID-19 register, Cochrane (library and COVID-19 register), and Scopus. Gray literature from MedRxiv, BioRxiv, AiXiv, and other preprint servers will be ensured through the inclusion of the Global Index Medicus database. No restriction will be set on language. The search for records will follow the PRISMA (Preferred Reporting Items for Systematic Reviews and Meta-Analysis) [44]. A PRISMA flow diagram will be used to present the workflow of how studies were included.

#### Manual Search

The search for useful articles will be conducted in Google scholar using the keywords of included articles, for objective 1, and the Cochrane COVID-19 Study Register for objective 2. We will also search manually through professional networks and other sources.

#### Citation Search

We hope to locate useful studies through the reference list of identified records by searching them online using their digital object identifiers in Crossref, DOI resolver, or through the journals in which they were published. Articles will also be identified through the reference list of suitable systematic reviews in the Cochrane Special Collections for COVID-19 Infection Control and Prevention.

#### Alerts and Generic Sources

Wherever necessary and possible, we will (1) set up email alerts in the electronic databases searched, (2) screen the citation manager’s generic suggestions as well as those from databases, and (3) screen database suggestions for similar articles.

### Search Strategy

#### Electronic Search Terms

Search terms will include “Qualitative”[Mesh], “Qualitative Study”[tiab], ”Mixed-method“[tiab], ”Interview“[Mesh], ”Attitude“[Mesh], ”Knowledge[Mesh]“, ”Practice“[Mesh], ”KAP“[tiab], ”Acceptance“[Mesh], ”Public Perception“[tiab], “Public Acceptance”[tiab], ”Public Attitude“[tiab], ”Compliance“[Mesh], “Adherence”[Mesh], ”Qualitative Research”[Mesh], “Explor*”[tiab], “COVID-19 Testing”[Mesh], “COVID-19 Nucleic Acid Testing “[Mesh], “Rapid Diagnostic Testing”[tiab], “Lateral Flow Tests”[tiab], “Nucleic Acid Testing”[Mesh], “Rapid test”[Mesh], Lateral Flow[tiab], “Mass Testing”[Mesh], “Mass Screening”[Mesh], “Diagnostic Test”[tiab], “Diagnostic Performance”[tiab], “Test Accuracy”[tiab], Test Performance”[tiab], “Universal Testing”[Mesh], “Universal Screening”[Mesh], “Point-of-Care Testing”[Mesh], “Point-of-Care”[tiab], “Point-of-Care Screening”[Mesh], “POC Testing”[Mesh], “Community Testing”[tiab], “Asymptomatic Infections”[Mesh], “Asymptomatic”[tiab], “Carrier State”[Mesh], “Asymptomatic Diseases”[Mesh], “COVID-19”[Mesh], “SARS-CoV-2”[Mesh], “2019-nCoV”[Mesh], “SARS-CoV-2 Variants”[Mesh], “VOC”[Mesh], “SARS-CoV-2 Variants”[Supplementary Concept], “RT-PCR”[tiab], “PCR”[Mesh], and “Molecular”[tiab]

#### Electronic Search Strategy

Records will be searched in databases highlighted under the electronic databases subsection. A date limit will be applied using electronic filters of search engines and set to 2020 and beyond. Searches will constantly be updated throughout the research period. A detailed search strategy in PubMed can be found in the study protocol [45]. The search strategy for each database can be seen in Table 2.

**Table 2:** Search strategy for each database.

Database and query order		Search query
**PubMed**		
	#1	(((((((((((((((((((((“Qualitative”[tiab]) OR (“Qualitative Study”[tiab])) OR (“Mixed-method”[tiab])) OR (“Mixed Method”[tiab])) OR (“Interview”[tiab])) OR (“Attitude”[MeSH])) OR (“Knowledge”[MeSH])) OR (“Practice”[tiab])) OR (“health personnel*”[tiab])) OR (“KAP”[tiab])) OR (“Acceptance”[tiab])) OR (“public acceptance”[tiab])) OR (“public perception”[tiab])) OR (“public attitude*”[tiab])) OR (“Compliance”[MeSH])) OR (“Patient Compliance”[MeSH])) OR (“Adherence”[tiab])) OR (“Patient adherence”[tiab])) OR (“Qualitative research”[MeSH])) OR (Explor*[tiab])) OR (“Health Knowledge, Attitudes, Practice”[MeSH])) OR (“Attitude of health personnel”[MeSH])
	#2	((((((((((“Point-of-Care Testing”[Mesh]) OR (“Point-of-Care”[tiab])) OR (“Point-of-Care Test*”[tiab])) OR (“Point-of-Care Detect*”[tiab])) OR (“Point-of-Care Screen*”[tiab])) OR (“Rapid Diagnostic Test*”[tiab])) OR (“Lateral Flow*”[tiab])) OR (“Mass Test*”[tiab])) OR (“Mass Screen*”[tiab])) OR (“Universal Test*”[tiab] OR “Universal Screen*”[tiab])) OR (“Rapid Test*”[tiab])
	#3	((“COVID-19 Testing”[Mesh]) OR (“COVID-19 testing”[tiab])) OR (“SARS-CoV-2 Testing”[tiab])
	#4	((((“SARS-CoV-2”[Mesh] OR (“COVID-19 Nucleic Acid Testing”[Mesh]) OR (“COVID-19 Nucleic Acid Testing”[tiab])) OR (“SARS-CoV-2 Nucleic Acid Testing”[tiab])) OR (“SARS-CoV-2 Nucleic Acid Detection”[tiab])) OR “SARS-CoV-2 variants” [Supplementary Concept])
	#5	#3 OR #4
	#6	(“PCR”[tiab]) OR (“RT-PCR”[tiab])
	#7	((((“Asymptomatic Infections”[Mesh]) OR (“Asymptomatic Infections”[tiab])) OR (“Asymptomatic”[tiab])) OR (“Asymptomatic Diseases”[tiab])) OR (“Carrier State”[tiab]) OR (“Carrier”[tiab])
	#8	#1 AND #2 AND #5 AND #7
	#9	#5 AND #6 AND #7
	#10	#6 AND #7
	#11	((((((((((“Point-of-Care Testing”[Mesh]) OR (“Point-of-Care”[tiab])) OR (“Point-of-Care Test*”[tiab])) OR (“Point-of-Care Detect*”[tiab])) OR (“Point-of-Care Screen*”[tiab])) OR (“Rapid Diagnostic Test*”[tiab])) OR (“Lateral Flow*”[tiab])) OR (“Mass Test*”[tiab])) OR (“Mass Screen*”[tiab])) OR (“Universal Test*”[tiab] OR “Universal Screen*”[tiab])) OR (“Rapid Test*”[tiab]) AND (((“Test accuracy”[tiab]) OR (“Test performance”[tiab])) OR (“Diagnostic accuracy”[tiab])) OR (“Diagnostic performance”[tiab])
	#12	#9 AND #11
	#13	#10 AND #11
	#14	#8 OR #12
	#15	#12 OR 13
	#16	#8 OR #15
**Cochrane**		
	#1	“Diagnostic” OR “Diagnostic test” OR “Diagnostic accuracy” OR “Diagnostic performance” OR “Test accuracy” in Title Abstract Keyword AND Mass test* OR Universal test* OR Universal screen* OR Point-of-care test* OR Rapid test* OR “PoC test*” OR Point-of-care* OR “Point-of-care test” OR “lateral flow” OR “lateral flow test” OR community test* in Title Abstract Keyword AND “Asymptomatic carrier” OR “Asymptomatic” OR SARS-CoV-2 OR “SARS-CoV-2 transmission” OR “Asymptomatic transmission” in Title Abstract Keyword AND “Qualitative study” OR Qualitative OR Mixed-method OR interview OR Acceptance OR Knowledge OR Attitude OR Practice OR Perspective OR Perception OR Adherence OR “Public acceptance” OR “Public perception” OR “Public attitude” OR “Public adherence” in Title Abstract Keyword - (Word variations have been searched)
	#2	“Diagnostic” OR “Diagnostic test” OR “Diagnostic accuracy” OR “Diagnostic performance” OR “Test accuracy” in Title Abstract Keyword AND Mass test* OR Universal test* OR Universal screen* OR Point-of-care test* OR Rapid test* OR Point-of-care* OR “Point-of-care test” OR “lateral flow” OR “lateral flow test” OR community test* in Title Abstract Keyword AND “RT PCR” OR RT-PCR OR “RT PCR test*” OR “RT-PCR analys*” OR “Molecular” in Title Abstract Keyword AND “Asymptomatic carrier” OR “Asymptomatic” OR SARS-CoV-2 OR “SARS-CoV-2 transmission” OR “Asymptomatic transmission” in Title Abstract Keyword - (Word variations have been searched)
	#3	#3= #1 OR #2
**EBSCOhost**		
	#1	AB (covid-19 or coronavirus or 2019-ncov or sars-cov-2 or cov-19 or pandemic or 2019 novel coronavirus or coronavirus disease) AND AB (rapid testing or antigene test or lateral flow test or mass test or universal test or mass screen or universal screen or point of care testing or poc or poct) AND AB (qualitative research or qualitative study or qualitative methods or interview or ethnographic or phenomenological)
	#2	AB (point of care testing or poc or poct ) OR AB mass screen* OR AB universal test* OR AB universal screen* OR AB lateral flow* OR AB rapid test* AND AB turnaround time AND AB diagnostic accuracy
	#3	AB (covid-19 or coronavirus or 2019-ncov or sars-cov-2 or cov-19) AND AB asymptomatic covid-19 NOT AB immune* NOT AB antibod* NOT AB immunoassay* NOT AB serolog* NOT AB seroprevalence NOT AB influenza NOT animal*
	#4	AB RT-PCR AND AB Asymptomatic*
	#5	S2 AND S3 AND S4
	#6	S1 OR S5
**Global Index Medicus**		
	#1	tw:((tw:(rapid test* OR lateral flow* OR point-of-care test* OR poc test* OR antigene test)) AND (tw:(covid-19 asymptomatic OR asymptomatic*)))
	#2	tw:((tw:(mass test*)) OR (tw:(universal screen*)) OR (tw:(universal test*)) OR (tw:(rapid test*)) OR (tw:(lateral flow*)) OR (tw:(point-of-care test*)) OR (tw:(poc test*)))
	#3	tw:((tw:(rt-pcr*)) AND (tw:(test accuracy OR test performance OR diagnostic accuracy OR diagnostic performance)))
	#4	tw:((tw:(covid-19 asymptomatic)) OR (tw:(asymptomatic*)))
	#5	#2 AND #3 AND #4
	#6	#1 OR #5
**Scopus**		
	#1	(TITLE-ABS-KEY ( covid-19 OR coronavirus OR 2019-ncov OR sars-cov-2 OR “novel coronavirus” ) AND TITLE-ABS-KEY (“rapid test*” OR “antigene test*” OR “lateral flow test*” OR “lateral flow” OR “mass test*” OR “universal test*” OR “point of care test*” OR “point-of-care”) AND TITLE-ABS-KEY (qualitative* OR mixed-method OR interview* OR perspective* OR knowledge OR attitude* OR practice OR perception OR “public perspective*” OR acceptance OR “public attitude”))
	#2	(TITLE-ABS-KEY (rapid AND test*) OR TITLE-ABS-KEY (lateral AND flow*) OR TITLE-ABS-KEY (mass AND test*) OR TITLE-ABS-KEY (universal AND test*) OR TITLE-ABS-KEY (point-of-care AND test*) OR TITLE-ABS-KEY (“point of care”))
	#3	(TITLE-ABS-KEY (covid-19) OR TITLE-ABS-KEY (sars-cov-2) OR TITLE-ABS-KEY (2019-ncov) AND TITLE-ABS-KEY (asymptomatic*))
	#4	(TITLE-ABS-KEY (rt-pcr* OR rt-pcr AND test*) AND TITLE-ABS-KEY (test AND accuracy OR diagnostic AND accuracy OR diagnostic AND performance))
	#5	#2 AND #3 AND #4
	#6	#1 OR #5

#### Search Peer Review

Search terms will be performed and verified by a second person to ensure search reproducibility. Both results were then compared, and any differences were resolved by modifying and rerunning the searches.

### Study Records

#### Data Management

Mendeley reference manager (Elsevier) and SWIFT-Review (Sciome) will be used to manage search records. SWIFT-Review workbench will be used to deduplicate, screen records, and code [46]. Data analysis will be conducted with the help of the SWIFT-Review and Stata package (Stata Corp).

#### Screening Process

We will start by identifying and removing duplicates in the first phase followed by the screening of titles and abstracts in the second phase, following the outlined inclusion and exclusion criteria. Screening of titles and abstracts will be conducted by 2 independent reviewers (PK and KA) while a third reviewer will serve as an arbitrator. If necessary, full articles will be read should it not be possible to screen records by title or abstract alone. The unavailability of full text will lead to exclusion.

#### Data Extraction

Data extraction for the first objective will be performed by the author leading the protocol development (MM) using SWIFT-Review. This will be done with the help of the SWIFT-Review Tag Browser and manually applied tags. Two reviewers (PK and KA) will independently extract data for the second objective using a customized data extraction template for RCTs and non-RCTs) [47]. The data form which can be accessed in Multimedia Appendix 1 aligns with the Methodological Expectations of Cochrane Intervention Reviews (MECIR) [48].

#### Management of References

A reference management software (Mendeley reference manager) will be used to manage records both for citations and for the generation of bibliography.

### Data Items

Data items to be extracted will include study title, year, author, country, study objective, conflicts of interest, funding, study design, population (age, sex, and ethnicity), setting, sample size, eligibility criteria, type of test, the time interval between the index and reference tests, the sample collected, statistical analysis, test status, time to results (turnaround time), test accuracy, and study limitations.

### Outcomes and Prioritization

The outcome of interest for the first objective is the test impact (patients, health personnel, and the general public). The outcome of the intervention for the second objective based on PICO is the status of SARS-CoV-2/COVID-19, which involves how well the test can identify those with the target condition and reject those without the conditions. Studies comparing the index and reference tests regarding the above will be prioritized.

### Risk-of-Bias Assessment

The assessment of the risk of bias of included studies will be conducted using the most relevant tools and based on study design [49]. Randomized controlled trials will be assessed using the revised version of the Cochrane Risk of Bias (RoB 2) assessment tool [50,51]. The items for risk-of-bias assessment using the RoB 2 tool will be organized into 5 domains including (1) bias due to randomization, (2) bias due to deviation from intended intervention, (3) bias due to missing data, (4) bias arising from outcome measurements, and (5) bias in the selection of reported results. Studies will be categorized into “low,” “some concerns,” and “high” for randomized controlled trials, as shown in Table S1 of Multimedia Appendix 2. Nonrandomized intervention studies will be assessed using the 8-item tool developed by the Evidence Project, suitable for a variety of study designs [52]. This tool assesses the risk of bias under 3 domains, including (1) study design, (2) representation of participants, and (3) comparison group of equivalence. Following the view not to attribute a summary risk-of-bias score for studies evaluated using the Evidence Project tool [52], the risk of bias of nonrandomized studies will be reported based on Table S2 of Multimedia Appendix 2.

### Metabias

Publication bias will be reduced through the inclusion of gray literature [53,54]. Additionally, in the event of a meta-analysis, publication bias will be assessed by inspecting the funnel plot. Any indication of missing data through funnel plot asymmetry will be adjusted using the “trim and fill” approach [55]. A positive difference between the corrected and uncorrected diagnostic accuracy values will be indicative of an overestimation of diagnostic accuracy due to missing data.

### Data Synthesis

#### Synthesis Procedure

Our synthesis approach for objective 1 to a greater part will take the form of a narrative synthesis [56,57]. Other studies have been found to use a similar method [58-61]. This will help maintain the contextual nature of the different mass testing options that will emanate from the literature. We will perform a meta-analysis for the second objective. We also anticipate possible heterogeneity in study design, study population, setting, and type of test, which may not make it possible to do a meta-analysis, in which case we will perform a synthesis without meta-analysis. Given the role played by context and system setup, we hope to capture both the quantitative and qualitative nature of diagnostic test studies.

#### Synthesis Framework

For outcomes under objective 1, the synthesis will follow four elements regarding the implementation of rapid mass testing, including (1) theoretical modeling, (2) preliminary synthesis (3), data interrelatedness, and (4) synthesis robustness [56]. Each of the aforementioned elements will be assessed using the most appropriate technique. An interpretative technique will be used to develop a theory about mass testing in relation to the outcomes of interest. The preliminary synthesis will be done using tabulation, textual description, clustering, and data translation. The relationship between data will be assessed using conceptual triangulation, case description, visual representation, and methodological triangulation. We will do a narrative synthesis to describe the outcomes of interest and other contextual factors. The synthesis robustness will be assessed through a critical reflection on the synthesis performed.

Concerning objective 2, we will perform a meta-analysis using the bivariate hierarchical logistic regression [62,63] in Stata, should there be a reasonable number of similar studies with the possibility of generating 2 x 2 tables. This multilevel model considers the correlation between sensitivity and specificity as well as variations in test performance between studies. If this is not possible, we will use positive and negative outcome events to pool event rates. We will inspect heterogeneity through direct observation of CIs on forest plots, where possible, and other graphs, as well as interstudy variability. The 95% CI binomial exact test will be used to report sensitivities and specificities for tests with single studies.

#### Data Presentation

The review findings will be presented using tables, figures, and case descriptions. Risk-of-bias assessment visualizations will be conducted using the robvis web application [64] where possible. Included studies will be organized by point-of-care setting using table rows and by study design, sample size, population, index test, index and reference tests interval, the sample collected, target gene, turnaround time, and accuracy, using table columns. Case summaries regarding outcomes of interest for the first objective (impact on patients, personnel, and the general public) will be summarized and presented according to the World Bank’s classification of countries by income for 2021-2022 [65]. Evidence of effect under the second objective will be presented using forest plots where possible.

### Quality of Evidence

The search strategy will follow the PRISMA search guidelines [66]. The quality of database search will be improved by searching beyond Medical Subject Headings (MeSH) terms by including “AND” and “OR” strings to the MeSH terms. Retained studies will be assessed for risk of bias using the most appropriate guidelines and checklists. The overall body of qualitative evidence in the first objective will be assessed using a critical reflection as highlighted [56], and the Grading of Recommendations, Assessment, Development, and Evaluation (GRADE) Confidence in the Evidence from Reviews of Qualitative research (GRADE-CERQual) [67]. If applicable, the GRADE will be used to assess the overall body of evidence regarding objective 2 [68,69]. This will consider the risk-of-bias assessments, precision in reported results, consistency, directness, and publication bias.

### Ethics and Dissemination

This study does not warrant any ethical approval. This protocol has been registered with the International Prospective Register of Systematic Reviews (PROSPERO; CRD42022283776) [45], in line with the standards for undertaking systematic reviews. Review results will be disseminated through conferences and their peer-reviewed publication in a relevant journal.

## Results

This protocol will be in line with the PRISMA protocol checklist [66]. We do not expect to have a reasonably high number of studies for rapid tests performed at point of care in LMICs in this review. Should this be the case, the results will be reported as 2 separate reviews and published in a relevant journal, as it might be unlikely to summarize all results in 1 report that robustly addresses both research questions. Following the above, the results of this review will be reported per (1) RAMESES (Realist and Metanarrative Evidence Syntheses: Evolving Standards) publication standards for meta-narrative reviews [70] regarding objective 1 and (2) the PRISMA checklist [71] or the Synthesis Without Meta-analysis in systematic reviews checklist [72], in the event that a meta-analysis was not possible, regarding objective 2.

**Figure 1:**
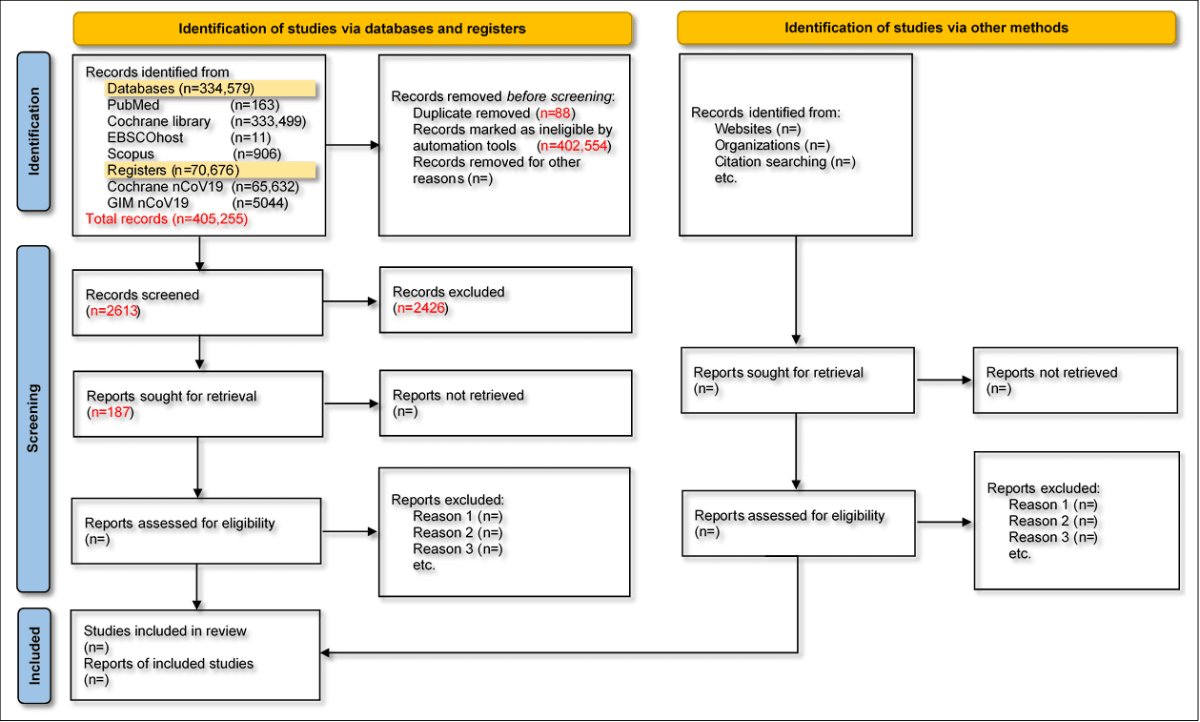
Preliminary PRISMA (Preferred Reporting Items for Systematic Reviews and Meta-Analyses) flow diagram. GIM: Global Index Medicus; nCoV19: COVID-19.

The second round of screening has been finalized. Figure 1 shows the search results from the different databases. We hope to finalize the synthesis by the end of February 2023 and to prepare the manuscript by April 2023.

## Discussion

### Summary

This protocol is based on the diagnostic usefulness and safety of rapid diagnostic tests in LMICs. A point-of-care test is one that can be delivered at the point of sample collection or near the patient, with results made available almost immediately. By “near the patient,” we refer to settings as specified by the Centers for Disease Control and Prevention [36] and the Medicines and Healthcare products Regulatory Agency [73]. A range of rapid tests esteemed to be useful in surveillance has been reported in the literature [18,74,75], broadly categorized into diagnostic, screening, and public health surveillance tests [76]. Moreover, 36 lateral flow devices believed to have the minimum performance requirements have been published by the UK government [37]. Their usefulness is however yet to be fully understood within the context of LMICs.

### Comparison With Prior Work

A search for reviews evaluating rapid point-of-care test against the gold standard on asymptomatic carriers in Cochrane library found 3 reviews [38,77,78]. Only 1 of the reviews [38] evaluated the diagnostic accuracy of rapid diagnostic tests. The review concluded that evidence for the testing of asymptomatic carriers was limited and highlighted the overriding need for the evaluation of rapid tests in the settings for which they are intended. This review will be the first, to the best of our knowledge, to address this knowledge gap in LMICs from a public health perspective.

Although rapid mass testing programs came under serious criticism early in the pandemic [79-82], it is still obvious that the symptom-based testing alone is not comprehensive enough as it failed to bring the virus under control prior to the approval of vaccines; this is partly because even the gold standard reverse-transcription polymerase chain reaction test is also prone to false negatives [83]. One possible way of ensuring that people are detected as soon as they become infected is by making a rapid mass testing regime available at point of care and service points [84].

### Strengths and Limitations

This protocol presents many strengths starting with its registration with PROSPERO. It is the first protocol that seeks to investigate both qualitative and quantitative evidence regarding the diagnostic safety of rapid mass tests in LMICs. The protocol’s specific objectives, search strategy, and reporting are all in line with the guidelines for the undertaking of systematic reviews.

### Conclusions

This review highlights the role of context in infection prevention and control using rapid mass testing. It flags the overriding need to involve users and providers in the evaluation of such tests in the settings for which they are intended. This will be the first review, to the best of our knowledge, to generate both qualitative and quantitative evidence regarding rapid mass testing specific to LMICs.

## Appendix

Multimedia Appendix 1 Data extraction form.

Multimedia Appendix 2 Risk-of-bias assessment tools.

## Abbreviations

CERQual: Confidence in the Evidence from Reviews of Qualitative research
GRADE: Grading of Recommendations Assessment, Development, and Evaluation
LMIC: low- and middle-income country
MeSH: Medical Subject Headings
PerSPEcTiF: Perspective, Setting, Phenomenon, Environment, Comparison, Timing, and Findings
PICO: population, intervention, comparison, outcome
PRISMA: Preferred Reporting Items for Systematic Reviews and Meta-Analysis
PROSPERO: International Prospective Register of Systematic Reviews
RAMESES: Realist and Metanarrative Evidence Syntheses: Evolving Standards

## Data Availability

Data sharing is not applicable to this article as no data sets were generated or analyzed during the current study.
