# Supplementary material for: A Metasynthesis and Meta-analysis of the Impact and Diagnostic Safety of COVID-19 Symptom Agnostic Rapid Testing in Low- and Middle-Income Countries: Protocol for a Systematic Review
Source: JMIR Res Protoc. 2023 Jan 5;12:e41132. doi: 10.2196/41132 (PMC9822567; doi:10.2196/41132)
Supplement: Multimedia Appendix 1 [file resprot_v12i1e41132_app1.docx]

A Meta-Synthesis and Meta-Analysis of The Impact and Diagnostic Safety of COVID-19 Symptom Agnostic Rapid Testing in LMICs: Protocol for Two Systematic Reviews

_________________________________________________________________________________________________________

# CUSTOMIZED Data Extraction Form

This form has been customized from the data extraction form for RCTs and Non-RCTs of the Cochrane Development, Psychosocial and Learning Problems Review Group[1] and as such complies with the Methodological Expectations of Cochrane Intervention Reviews (MECIR)[2].

# General Information

| Reviewer Date:  *Date data was extracted* | Cliquez ici pour entrer une date. |
| --- | --- |
| Reviewer ID:  *ID of person extracting data* | Choisissez un élément. |
| Study ID:  *(e.g., Higgins, 2020)* |  |
| Study Title:  *Input full title of study* |  |
| Ethical Approval:  *Approval details provided* | Choisissez un élément. |
| Funding:  *Input funding details* |  |
| Conflict of Interest:  *Was the CoI clearly stated?* | Choisissez un élément. |
| Study limitations:  *Were these reported?* | Choisissez un élément. |

# Application of Eligibility Criteria

| **Item Description** | **Decision criteria** | **Decision** | **Eligible** | | |
| --- | --- | --- | --- | --- | --- |
|  |  |  | **Yes** | **No** | **Unclear** |
| Study Design: | Cohort cross-sectional, cross-sectional = Yes  Case control cross-sectional = Yes  Mixed-method or Qualitative = Yes  Longitudinal cohort or case control = No | Choisissez un élément. |  |  |  |
| Study Environment: | Low-income, lower-middle & upper middle-income = Yes  High-income = No | Choisissez un élément. |  |  |  |
| Participants: | Unknown disease status and asymptomatic subjects = Yes  COVID-19 and symptomatic patients = No | Choisissez un élément. |  |  |  |
|  | If study included both symptomatic and asymptomatic subjects, was there a subgroup analysis for asymptomatic subjects | Choisissez un élément. |  |  |  |
| Sample Analysis: | Non lab-based sample analysis = Yes  Lab-based sample analysis = No | Choisissez un élément. |  |  |  |
| Index Test: | Rapid Antigen Test = Yes  Point-of-Care Test = Yes  Lateral Flow Test = Yes  Serology/antibody rapid test = No  Non sample-based (imaging) = No | Choisissez un élément. |  |  |  |
| Comparator:  *Reference test used* | RT-PCR test = Yes  Other tests other than RT-PCR = No  “N/A” if Mixed method or Qualitative | Choisissez un élément. |  |  |  |
| Target Condition: | SARS-CoV-2 related study = Yes  Non SARS-CoV-2 related study = No | Choisissez un élément. |  |  |  |
| Time between tests: | Simultaneously/almost the same time = Yes  Considerable time difference = No  “N/A” if Mixed method of Qualitative | Choisissez un élément. |  |  |  |
| Outcome: | Diagnostic accuracy = Yes  Prognostic accuracy = No  Predictive accuracy = No  Knowledge, Attitude, Perception = Yes  Impact = Yes | Choisissez un élément. |  |  |  |
| INCLUSION DECISION |  | Choisissez un élément. |  |  |  |
| Reasons if excluded | *Note: No subgroup analysis for asymptomatic subjects | | | | |

***Only continue data extraction beyond this level for Included Studies**

# INCLUDED STUDY CHARACTERISTICS

| Study location:  *Specify country of study* |  |
| --- | --- |
| Study Objective:  *Input the study’s specific objective* |  |
| Sample size: *Total:* |  |
| *Men:* |  |
| *Women:* |  |
| *Mean age:* |  |
| *Ethnicity:* |  |
| Study subjects: | Choisissez un élément. |
| Participant selection:  *How were participants selected?* | Choisissez un élément. |
| Eligibility Criteria:  *Where eligibility criteria specified?* | Choisissez un élément. |
| Study Setting:  *Specify testing site* | Choisissez un élément. |
| Sample collected:  *Specify type of sample collected* | Choisissez un élément. |
| Type of Test (Intervention):  *Device (LFD) details of index test* |  |
| Comparator:  *Device (LFD) details of reference test* |  |
| Time interval:  *Specify time between the two tests* |  |
| Outcome (Test Status): |  |
| TP: |  |
| TN: |  |
| FP: |  |
| FN: |  |
| Data analysis:  *Statistical approach used* |  |
| Time to results:  *Index test turnaround time* |  |

# References

1. The Cochrane Collaboration. Data extraction forms. Cochrane Developmental, Psychosocial and Learning Problems. https://dplp.cochrane.org/data-extraction-forms. Published 2014. Accessed January 6, 2022.

2. Churchill R, Lasserson T, Chandler J, Tovey D, Thomas J, Flemyng E HJ. Standards for the reporting of new Cochrane Intervention Reviews. In: *Higgins JPT, Lasserson T, Chandler J, Tovey D, Thomas J, Flemyng E, Churchill R. Methodological Expectations of Cochrane Intervention Reviews.* Cochrane: London; 2021. https://community.cochrane.org/book_pdf/580. Accessed January 6, 2022.
