# Supplementary material for: A Metasynthesis and Meta-analysis of the Impact and Diagnostic Safety of COVID-19 Symptom Agnostic Rapid Testing in Low- and Middle-Income Countries: Protocol for a Systematic Review
Source: JMIR Res Protoc. 2023 Jan 5;12:e41132. doi: 10.2196/41132 (PMC9822567; doi:10.2196/41132)
Supplement: Multimedia Appendix 2 [file resprot_v12i1e41132_app2.docx]

A Meta-Synthesis and Meta-Analysis of The Impact and Diagnostic Safety of COVID-19 Symptom Agnostic Rapid Testing in LMICs: Protocol for Two Systematic Reviews

_________________________________________________________________________________________________________

### **S1: Risk of Bias Assessment of Randomized Controlled Trials**

|  | **Issues to be addressed** | **Response** | | | | |
| --- | --- | --- | --- | --- | --- | --- |
|  |  |  |  |  |  |  |
| **Bias arising from the randomization process** | | | | | | |
|  | Whether the allocation sequence was random | Y | PY | PN | N | NI |
|  | Whether the allocation sequence was adequately concealed | Y | PY | PN | N | NI |
|  | Whether baseline differences between intervention groups suggest a problem with the randomization process | Y | PY | PN | N | NI |
|  | Domain risk-of-bias judgment2 |  | | | | |
| **Bias due to deviations from intended interventions** | | | | | | |
|  | Whether participants were aware of their assigned intervention during the trial | Y | PY | PN | N | NI |
|  | Whether carers and people delivering the interventions were aware of participants’ assigned intervention during the trial | Y | PY | PN | N | NI |
|  | If important non-protocol interventions were balanced across intervention groups | Y | PY | PN | N | NI |
|  | If failures in implementing the intervention as per protocol could have affected the outcome | Y | PY | PN | N | NI |
|  | If study participants adhered to the assigned intervention regimen | Y | PY | PN | N | NI |
|  | If an appropriate analysis was used to estimate the effect of adhering to the intervention | Y | PY | PN | N | NI |
|  | Domain risk-of-bias judgment |  | | | | |
| **Bias due to missing outcome data** | | | | | | |
|  | Whether data for the outcome were available for all, or nearly all, participants randomized | Y | PY | PN | N | NI |
|  | If there was evidence that the result was not biased by missing outcome data | Y | PY | PN | N | NI |
|  | If the missing outcome data was likely to depend on its true value | Y | PY | PN | N | NI |
|  | Domain risk-of-bias judgment |  | | | | |
| **Bias in measurement of the outcome** | | | | | | |
|  | Whether the method used in measuring the outcome was inappropriate | Y | PY | PN | N | NI |
|  | Whether measurement or ascertainment of the outcome could have differed between intervention groups | Y | PY | PN | N | NI |
|  | Whether outcome assessors were aware of the intervention received by study participants | Y | PY | PN | N | NI |
|  | If the assessment of the outcome was likely to have been influenced by knowledge of intervention received | Y | PY | PN | N | NI |
|  | Domain risk-of-bias judgment |  | | | | |
| **Bias in selection of the reported result** | | | | | | |
|  | Whether data was analyzed in accordance with a pre-specified plan that was finalized before unblinded outcome data were available for analysis | Y | PY | PN | N | NI |
|  | Whether the numerical result being assessed is likely to have been selected, on the basis of the results, from multiple outcome measurements within the outcome domain | Y | PY | PN | N | NI |
|  | Whether the numerical result being assessed is likely to have been selected, on the basis of the results, from multiple analyses of the data | Y | PY | PN | N | NI |
|  | Domain risk-of-bias judgment |  | | | | |
| **Overall risk-of-bias judgment** | | | | | | |
|  | Risk-of-bias judgment2 |  | | | | |

1Y=Yes, PY=Probably yes, PN=Probably no, N=No, NI=No information

2Low=Judged to be at low risk of bias for all domains

2Some concerns=Raises concerns in at least one domain but not at high risk for any domain

2High=High risk of bias in at least one domain or has some concerns for multiple domains

### **S2: Risk of Bias Assessment of Non-Randomized Studies**

|  | **Assessment Items** | **Responses** | | | |
| --- | --- | --- | --- | --- | --- |
|  |  |  |  |  |  |
| **Study design** | | | | | |
|  | Cohort | Y | N | NA | NR |
|  | Control or comparison group | Y | N | NA | NR |
|  | Pre/post-intervention data | Y | N | NA | NR |
|  | **Risk of bias judgment** |  |  |  |  |
| **Participant representativeness** | | | | | |
|  | Random assignment of participants to the intervention | Y | N | NA | NR |
|  | Random selection of participants for assessment | Y | N | NA | NR |
|  | Follow-up rate of 80% or more | Y | N | NA | NR |
|  | **Risk of bias judgment** |  |  |  |  |
| **Equivalence of comparison groups** | | | | | |
|  | Comparison groups equivalent on sociodemographics | Y | N | NA | NR |
|  | Comparison groups equivalent at baseline on outcome measures | Y | N | NA | NR |
|  | **Risk of bias judgment** |  |  |  |  |
|  |  |  |  |  |  |

1Y=Yes, N=No, NA=Not applicable, NR=Not reported
